# Dietary diversity and diet quality with gestational weight gain and adverse birth outcomes, results from a prospective pregnancy cohort study in urban Tanzania

**DOI:** 10.1111/mcn.13300

**Published:** 2021-12-14

**Authors:** Jiaxi Yang, Molin Wang, Deirdre K. Tobias, Janet W. Rich‐Edwards, Anne‐Marie Darling, Ajibola I. Abioye, Ramadhani A. Noor, Isabel Madzorera, Wafaie W. Fawzi

**Affiliations:** ^1^ Department of Epidemiology Harvard T. H. Chan School of Public Health Boston Massachusetts USA; ^2^ Department of Biostatistics Harvard T. H. Chan School of Public Health Boston Massachusetts USA; ^3^ Department of Nutrition Harvard T. H. Chan School of Public Health Boston Massachusetts USA; ^4^ Division of Preventive Medicine, Department of Medicine Brigham and Women's Hospital Boston Massachusetts USA; ^5^ Department of Global Health and Population Harvard T.H. Chan School of Public Health Boston Massachusetts USA; ^6^ United Nations Children's Fund (UNICEF) Dar es Salaam Tanzania

**Keywords:** birth outcomes, gestational weight gain, low birth weight, maternal diet, minimum dietary diversity for women, preterm birth, prime diet quality score, Tanzania

## Abstract

Healthy maternal diets during pregnancy are an important protective factor for pregnancy‐related outcomes, including gestational weight gain (GWG) and birth outcomes. We prospectively examined the associations of maternal dietary diversity and diet quality, using Minimum Dietary Diversity for Women (MDD‐W) and Prime Diet Quality Score (PDQS), with GWG and birth outcomes among women enrolled in a trial in Tanzania (*n* = 1190). MDD‐W and PDQS were derived from a baseline food frequency questionnaire. Women were monthly followed until delivery, during which weight was measured. GWG was classified based on the 2009 Institute of Medicine guidelines. Adverse birth outcomes were classified as low birth weight (LBW), small for gestational age, large for gestational age, and preterm birth. 46.2% participants had MDD‐W ≥ 5. Mean score of PDQS was 23.3. Maternal intakes of nuts, poultry, and eggs were low, whereas intakes of sugar‐sweetened beverages and refined grains were high. MDD‐W was not associated with GWG or birth outcomes. For PDQS, compared to the lowest tertile, women in the highest tertile had lower risk of inappropriate GWG (risk ratio [RR] = 0.93, 95% confidence interval [CI]: 0.87–1.00). Women in the middle tertile group of PDQS (RR = 0.72, 95% CI: 0.51–1.00) had lower risk of preterm birth. After excluding women with prior complications, higher PDQS was associated with lower risk of LBW (middle tertile: RR = 0.55, 95% CI: 0.31–0.99, highest tertile: RR = 0.52, 95% CI: 0.29–0.94; continuous per SD: RR = 0.77, 95% CI: 0.60–0.99). Our findings support continuing efforts to improve maternal diet quality for optimal GWG and infant outcomes among Tanzanian women.

## Key messages

• Maternal diets are a key modifiable determinant of gestational weight gain (GWG) and birth outcomes.
• We observed suboptimal intakes of healthy proteins and fats and high intakes of refined grains and sugar‐containing foods among well‐nourished pregnant women in urban Tanzania.
• This study found that higher‐quality maternal diets were associated with lower risks of inappropriate GWG, low birth weight, and preterm birth.
• This study supports the importance of high maternal diet quality and continuing efforts to promote well‐balanced maternal diets with avoiding both under‐ and over‐nutrition for optimal pregnancy outcomes among Tanzanian populations.

## 1 INTRODUCTION

Maternal diet is a modifiable risk factor for poor birth outcomes (Abu‐Saad & Fraser, 2010; Imdad & Bhutta, 2012). Poor maternal diets during pregnancy may result in poor nutrition status and adverse birth outcomes, including low birth weight (LBW), preterm birth, and intrauterine growth restriction (Abu‐Saad & Fraser, 2010). Adverse birth outcomes are associated with increased risk of neonatal complications and long‐term consequences for the infant, including cognitive impairment, stunting, and childhood obesity (Black et al., 2008; Imdad & Bhutta, 2012; Sebire et al., 2001).

For women during pregnancy, micronutrient adequacy is essential in preventing malnutrition as well as the adverse birth outcomes related to malnutrition (Ramakrishnan, 2002). Studies conducted in low‐ and middle‐income countries (LMIC) have documented the importance of micronutrients, such as folate, iron, zinc, and other essential vitamins and minerals, for preventing adverse birth outcomes, including LBW, small for gestational age (SGA), and stillbirths (Fawzi et al., 2007; Gernand et al., 2016; Grieger & Clifton, 2014; Ramakrishnan, 2002; Zerfu & Ayele, 2013). Maternal diets with diverse sources of foods are more likely to provide sufficient micronutrients required for the mother's health and the development of the fetus (Gernand et al., 2016). Limited evidence from prospective studies in Sub‐Saharan Africa (SSA) have supported the associations between maternal dietary diversity assessed by Minimum Dietary Diversity for Women (MDD‐W) and lower risks of pregnancy outcomes, including LBW, SGA, stillbirth, and preterm birth (Madzorera et al., 2020; Nsereko et al., 2020; Zerfu et al., 2016).

While consuming foods of diverse sources may benefit micronutrient sufficiency, the choice of foods is relevant to maternal diet quality and its influence on birth outcomes (Abu‐Saad & Fraser, 2010; Chia et al., 2019). High‐quality diet emphasizes fruits and vegetables, whole grains, healthy fats and proteins, and low intakes from sodium or added sugar (Chiuve et al., 2012). A low‐quality maternal diet with proportionally high intake of unhealthy foods not only fails to provide proper nutrition required during pregnancy but may contribute to maternal obesity, inflammation, and ultimately pregnancy or birth complications (King, 2006; Sen et al., 2015). Using Prime Diet Quality Score (PDQS) assessing overall diet quality (Fung et al., 2018; Rifas‐Shiman et al., 2001), studies have supported the role of maternal diet quality on pregnancy outcomes, including preterm birth, LBW, and stillbirth in countries of SSA (Madzorera et al., 2020), and gestational diabetes mellitus in a prospective study in the United States (Gicevic et al., 2018).

Given the importance of maternal diets for birth outcomes and the long‐term health consequences of these outcomes, examining the contribution of maternal diets in preventing adverse birth outcomes is important for SSA countries, where rates of these outcomes remain high (Katz et al., 2013). Furthermore, recently, some SSA countries are undergoing transitions from low‐ to middle‐income status, with better food security and improved access to diverse foods (Steyn & McHiza, 2014; Vorster et al., 2011). However, the availability of diverse foods could also result in a shift from traditional to Western dietary patterns, with increasing consumption of high‐calorie, refined and fast foods with low micronutrient density, leading to poor maternal diet quality and consequently obesity and obesity‐related pregnancy complications (Lindsay et al., 2012; Popkin et al., 2012; Wrottesley et al., 2017). Thus, it is important to examine both maternal dietary diversity and quality in recent SSA populations experiencing these nutrition transitions.

Examining the associations between dietary patterns using dietary scores and adverse birth outcomes is useful for providing specific dietary advice in practice, particularly for high‐risk populations (Hu, 2002). In addition, since gestational weight gain (GWG) is a key mediator for maternal diet and birth outcomes (Parker et al., 2019), as well as a strong risk factor for other pregnancy complications on its own (Institute of Medicine & National Research Council 2009), it is also meaningful to examine the role of maternal diet on GWG (Figures S1 and S2). A few studies in SSA have examined MDD‐W and PDQS with birth outcomes, using 24‐h recalls (Madzorera et al., 2020; Zerfu et al., 2016). One study in Rwanda with limited sample size examined MDD‐W using food frequency questionnaire (FFQ) and risk of preterm birth (Nsereko et al., 2020). Thus, evidence on MDD‐W and PDQS characterized by FFQ with respect to other birth outcomes is sparse. Furthermore, the associations of MDD‐W and PDQS with GWG remained largely unexplored in SSA populations.

This study prospectively examined maternal dietary diversity and quality using MDD‐W and PDQS, respectively, derived from FFQs, and their associations with GWG and adverse birth outcomes, including LBW, SGA, large for gestational age (LGA), and preterm birth in a healthy pregnancy cohort from urban Tanzania.

## 2 METHODS

### 2.1 Study population

We used data from a randomized clinical trial conducted among pregnant women in urban Tanzania. Details of this study has been described elsewhere (Etheredge et al., 2015). Briefly, from September 2010 to October 2012, a randomized placebo‐controlled trial of iron supplements was conducted in Dar es Salaam, Tanzania. Participants were screened and enrolled at antenatal care clinics. Women were eligible if they were iron‐replete, nonanemic, HIV‐uninfected, primigravidae or secundigravidae, and at or before 27 weeks of gestation at recruitment. The study enrolled 1500 pregnant women who were subsequently randomized to receive either a daily dose of 60 mg iron or placebo from the time of enrollment until delivery. At baseline, participants completed a sociodemographic and reproductive health questionnaire, dietary assessment using an FFQ, as well as a full clinical examination. Women subsequently attended monthly antenatal visits until delivery to receive standard of care (Etheredge et al., 2015). Pregnancy outcomes were recorded by on‐site midwives at time of delivery.

For the current investigation, we excluded women with missing baseline FFQ (*n *= 9) or implausible total energy intake (<500 or ≥3500 kcal, *n *= 31), with one weight measurement only during the follow‐up period (*n *= 206), with unknown gestational age at delivery (*n *= 22) or delivery outcomes (*n *= 15), or with twin births (*n *= 27), resulting in a final study sample of 1190 participants.

Ethical approval for the study was provided by the Harvard School of Public Health Human Subjects Committee, the Muhimbili University of Health and Allied Sciences Research and Publications Committee, and the Tanzania's National Institute for Medical Research. Written informed consent was obtained from all women for their participation in the study.

### 2.2 Exposure assessment

The primary exposures of interest were maternal dietary diversity and diet quality, measured by two dietary scores, MDD‐W and PDQS, respectively. At baseline, research assistants administered a semi‐quantitative FFQ inquiring how often, on average, a participant had consumed a specified amount of common foods in the preceding month. The FFQ was developed to reflect the local dietary patterns in the general population in Tanzania and has been employed in previous studies conducted in the region (Abioye et al., 2015; Lukmanji et al., 2013). It included 108 individual food items grouped under foods eaten and food eaten alone and/or mixed in a meal and 11 ingredients (Abioye et al., 2015). For each food item, participant was asked to select the option that would best reflect her intake in the past month: never (0 times in a month), 1–3 times per month, 1 time per week, 2–4 times per week, 5–6 times per week, 1 time per day, 2–3 times per day, 4–5 times per day, or 6+ times per day. From the FFQ, daily consumptions of macronutrients, micronutrients, and total energy intake (kcal) were estimated using the Tanzania Food Composition Tables (Lukmanji et al., 2008). Based on the reported frequency, we derived serving/day for each individual food item (0 serving/day for “never,” 0.07 serving/day for “1–3 times per month,” 0.14 serving/day for “1 time per week,” 0.43 serving/day for “2–4 times per week,” 0.79 serving/day for “5–6 times per week,” 1 serving/day for “1 time per day,” 2.5 servings/day for “2–3 times per day,” 4.5 servings/day for “4–5 times per day,” and 6 servings/day for “6+ times per day” (Table S1) (Rosner & Gore, 2001).

### 2.3 Minimum Dietary Diversity for Women

MDD‐W was derived based on the baseline FFQ. Details on MDD‐W have been described elsewhere (Food and Agriculture Organization [FAO], 2021). Briefly, MDD‐W was originally developed by FAO as a population‐level dichotomous indicator as a proxy measure of micronutrient adequacy for women of reproductive age living in resource‐limited settings. The indicator is defined as consumption of at least five out of ten defined food groups on the previous day. It includes the following ten food groups: (1) starchy staples, (2) beans and peas, (3) nuts and seeds, (4) dairy, (5) flesh foods (meat, fish), (6) eggs, (7) vitamin A‐rich dark green vegetables, (8) other vitamin A‐rich fruits and vegetables, (9) other vegetables, and (10) other fruits. Individual food items in the FFQ were grouped into their corresponding MDD‐W food group. A woman was considered as having consumed the foods from a food group (+1 point) if she reported intake from any of the food(s) under that food group with a combined frequency of one time per day or higher. We followed the same MDD‐W grouping methods outlined in the previous study by Madzorera et al. Specifically, for mixed dishes, a dish was grouped into one of the ten food groups based on the main component of the dish; maize and kidney beans were grouped under starchy staples and beans and peas, respectively (Madzorera et al., 2020). Points were summed for the ten MDD‐W food groups. MDD‐W ranged from 0 to 10, with ≥5 points considered as meeting the minimum dietary diversity (FAO, 2021).

### 2.4 Prime Diet Quality Score

The same baseline FFQ was used to derive the PDQS for maternal diet quality. Details on PDQS have been described elsewhere (Fung et al., 2018; Gicevic et al., 2018). Briefly, the PDQS is composed of fourteen healthy food groups (dark green vegetables, cruciferous vegetables, carrots, other vegetables, whole citrus fruits, other fruits, legumes, nuts and seeds, poultry, fish, eggs, whole grains, liquid vegetable oils, and low‐fat dairy) and seven unhealthy food groups (potatoes, red meat, processed meat, refined grains and baked goods, sugar‐sweetened beverages, fried foods eaten away from home, and deserts and ice cream). Individual food items were grouped into their corresponding PDQS food group. Similar to the MDD‐W grouping, only the main component of a mixed dish was assigned to the appropriate PDQS food group; other vitamin A‐rich fruits and vegetables (pumpkin, mango, and passion fruit) were additionally included into the group of carrots (Madzorera et al., 2020).

Daily serving(s) for all the food items included in each food group were summed and then multiplied by 7 to represent the total weekly serving(s) for that particular food group. Depending on the food group (healthy vs. unhealthy) and the summed weekly food serving(s), a score for each food group was assigned (healthy food groups: 0 points for 0–1 servings/week, 1 point for 2–3 servings/week, and 2 points for 4+ servings/week; unhealthy food groups: 2 points for 0–1 servings/week, 1 point for 2–3 servings/week, and 0 points for 4+ servings/week). The scores were then summed to give the total PDQS score. Due to their limited consumption in Tanzania, low‐fat dairy from the healthy food groups and processed meat from the unhealthy food groups were not collected in the FFQ. As a result, all participants received 0 point for low‐fat dairy and 2 points for processed meat (Madzorera et al., 2020). PDQS had a range of 0–42, with a higher score indicating overall higher diet quality.

### 2.5 Outcome assessment

#### 2.5.1 Measurement and characterization of GWG

Participants’ weight (kg) was measured at baseline and at monthly antenatal visits by trained study nurses using a calibrated weight scale. For the outcome of GWG, we defined appropriate GWG based on the 2009 IOM guidelines (weekly GWG rate in the second and third trimesters: 0.44–0.58 kg/week for body mass index [BMI] < 18.5 kg/m^2^, 0.35–0.50 kg/week for BMI between 18.5 and 25 kg/m^2^, 0.23–0.33 kg/week for BMI between 25 and 30 kg/m^2^, and 0.17–0.27 kg/week for BMI ≥ 30 kg/m^2^) (IOM & NRC, 2009). The IOM guidelines on GWG required the knowledge of pre‐pregnancy BMI, which was not available in the original study. Given the overall distribution of available maternal weight measures, we imputed pregnancy weight at 14 weeks of gestation using mixed‐effects models with polynomial terms for gestational age, and statistical results suggested good model fit (Yang et al., 2021). Based on the imputed weight and the height measured at baseline, BMI status at the end of the first trimester was derived accordingly. The weekly rate of GWG (kg/week) was derived by calculating the difference between the first measured weight in the second trimester and the last measured weight before delivery and dividing that by the number of weeks between the two measures. Based on the calculated GWG rate, the BMI status at 14 weeks of gestation, and the BMI‐specific recommended range for GWG rate provided by the IOM (IOM & NRC, 2009), three binary GWG outcomes were created: inadequate GWG (GWG rate below the recommended range), excessive GWG (GWG rate above the recommended range), and inappropriate GWG (GWG rate either below or above the recommended range).

### 2.6 Adverse birth outcomes

For pregnancies resulting in live births, the following outcome characteristics were available in the study: gestational age at delivery, infant sex, and infant birthweight. We calculated LBW (birthweight < 2.5 kg), SGA and LGA (gender‐specific birth weight below 10th percentile and above 90th percentile respectively for babies of the same gestational age according to the INTERGROWTH‐21st reference chart) (Villar et al., 2014), and preterm birth (gestational age at delivery <37 weeks).

### 2.7 Statistical analysis

In the main analysis, we evaluated the associations between the two dietary scores, MDD‐W and PDQS, and GWG and adverse birth outcomes. For each dietary score, we created tertile groups, with the lowest tertile group set as the reference group; and a continuous score divided by 1 SD was additionally evaluated. MDD‐W with binary levels was additionally modeled based on the conventional cut‐off for meeting minimum dietary diversity (i.e., ≥5 and <5, with <5 set as the reference group). For outcome variables, GWG and adverse birth outcomes were modeled as binary outcomes (yes, no). We used multivariable Poisson regression with a sandwich variance estimator to calculate risk ratio (RR) and 95% confidence interval (CI) (Zou, 2004).

Covariates hypothesized a priori as potential confounders were adjusted for in the models. These included baseline age (years), gestational age (weeks), BMI (kg/m^2^), season (dry [December–March], long rains [April–May], harvest [June–September], short rains [October–November]) (Lawrence et al., 1987; Madzorera et al., 2020), primigravida status (yes, no), marital status (married or cohabitating, other), treatment status (treatment, placebo), education (0–4, 5–7, 8–11, >11 years), occupation (unemployed, unskilled/informal, skilled, other), and history of prior complications (yes if any past complication in cardiovascular disease, high blood pressure, diabetes, weight loss in the previous year, or ever having a LBW infant or non‐live birth among non‐primigravida). Since energy intake was a potential mediator, we did not adjust for it in the models. To address the potential residual confounding due to pre‐existing conditions, we repeated the analyses excluding women with prior history of complications (excluded *n *= 186). All analyses were conducted using SAS statistical software (version 9.4; SAS Institute Inc.). All statistical tests were two‐sided, with *p* values <0.05 considered statistically significant.

## 3 RESULTS

### 3.1 MDD‐W, GWG, and adverse birth outcomes

Our study included 1190 study participants, with mean age of 24.1 years and mean gestational age of 18.0 weeks at baseline (Table 1). For the overall MDD‐W profile in the study population, the mean score was 4.2 (SD = 1.9), and 46.2% (*n *= 550) met the minimum dietary diversity criteria defined by MDD‐W ≥ 5 (Table 2). Across the ten MDD‐W food groups, consumptions of starchy staples, meat, poultry, fish, vegetables, and fruits were high, whereas consumptions of nuts and seeds, dairy, and eggs were low. MDD‐W was strongly correlated with energy intake (Spearman *r* = 0.72) and PDQS (Spearman *r* = 0.52) (Table 2).

**Table 1:** Baseline population characteristics by tertiles of MDD‐W and PDQS scores (*n *= 1190)

	MDD‐W^a^			PDQS^b^		
Tertile group (range) *n*	Tertile 1 (1, 3)	Tertile 2 (4, 5)	Tertile 3 (6, 9)	Tertile 1 (10, 21)	Tertile 2 (22, 24)	Tertile 3 (25, 31)
	*n* = 437	*n* = 414	*n* = 339	*n* = 349	*n* = 415	*n* = 426
Baseline age (years), mean (SD)	24.0 (4.1)	24.0 (4.2)	24.3 (4.3)	24.1 (4.3)	24.1 (4.0)	24.1 (4.3)
Weight at baseline (kg), mean (SD)	59.5 (11.2)	60.7 (12.1)	59.6 (11.9)	60.3 (12.2)	60.5 (11.9)	59.1 (11.0)
Height at baseline (cm), mean (SD)	155.9 (6.0)	156.5 (6.2)	156.1 (5.9)	156.6 (6.4)	156.6 (5.9)	155.5 (5.8)
BMI at baseline (kg/m^2^), mean (SD)	24.5 (4.6)	24.7 (4.5)	24.4 (4.6)	24.6 (5.0)	24.7 (4.6)	24.4 (4.3)
BMI at 14 weeks of gestation (kg/m^2^), mean (SD)	23.9 (4.4)	24.2 (4.4)	23.9 (4.4)	24.0 (4.7)	24.1 (4.4)	23.8 (4.0)
Gestational age at baseline (weeks), mean (SD)	17.9 (4.5)	17.8 (4.2)	18.2 (4.1)	17.8 (4.5)	17.9 (4.3)	18.1 (4.1)
Season at baseline, *n* (%)						
Dry (December–March)	141 (32.3)	127 (30.7)	113 (33.3)	117 (33.5)	131 (31.6)	133 (31.2)
Long rains (April–May)	89 (20.4)	73 (17.6)	61 (18.0)	61 (17.5)	86 (20.7)	76 (17.8)
Harvest (June–September)	90 (20.6)	158 (38.2)	127 (37.5)	97 (27.8)	128 (30.8)	150 (35.2)
Short rains (October–November)	117 (26.8)	56 (13.5)	38 (11.2)	74 (21.2)	70 (16.9)	67 (15.7)
Married/cohabitating, *n* (%)	338 (77.4)	320 (77.3)	291 (85.8)	278 (79.7)	330 (79.5)	341 (80.1)
Treatment status (iron), *n* (%)	227 (52.0)	203 (49.0)	155 (45.7)	177 (50.7)	206 (49.6)	202 (47.4)
Occupation, *n* (%)						
Unemployed	204 (46.7)	197 (47.6)	159 (46.9)	161 (46.1)	195 (47.0)	204 (47.9)
Unskilled/informal	156 (35.7)	114 (27.5)	92 (27.1)	111 (31.8)	119 (28.7)	132 (31.0)
Skilled	69 (15.8)	92 (22.2)	69 (20.4)	63 (18.1)	94 (22.7)	73 (17.1)
Other	8 (1.8)	11 (2.7)	19 (5.6)	14 (4.0)	7 (1.7)	17 (4.0)
Primigravida, *n* (%)	261 (59.7)	238 (57.5)	187 (55.2)	199 (57.0)	249 (60.0)	238 (55.9)
Education status, *n* (%)						
0–4 years	19 (4.4)	20 (4.8)	20 (5.9)	14 (4.0)	19 (4.6)	25 (6.1)
5–7 years	238 (54.5)	230 (55.6)	151 (44.5)	177 (50.7)	206 (49.6)	236 (55.4)
8–11 years	123 (28.2)	112 (27.1)	98 (28.9)	112 (32.1)	122 (29.4)	99 (23.2)
>11 years	57 (13.0)	52 (12.6)	70 (20.7)	46 (13.2)	68 (16.4)	65 (15.3)
History of prior complications, *n* (%)^c^	55 (12.6)	74 (17.9)	57 (16.8)	50 (14.3)	62 (14.9)	74 (17.4)
*Major nutrients and food intakes, mean (SD)*						
Total energy intake (kcal/day)	1748 (539)	2467 (599)	3079 (698)	2029 (746)	2309 (774)	2730 (758)
Carbohydrate (% energy)^d^	53.7 (7.3)	51.5 (7.0)	49.9 (5.9)	53.2 (7.2)	52.0 (7.3)	50.5 (6.3)
Protein (% energy)	13.7 (3.2)	14.3 (2.8)	15.1 (2.5)	13.2 (3.0)	14.4 (3.0)	15.1 (2.6)
Fat (% energy)	23.7 (6.2)	34.2 (6.0)	35.0 (5.1)	33.5 (6.3)	33.6 (6.2)	34.3 (5.3)
Vegetable (serving/day)	1.4 (0.8)	2.8 (1.4)	4.2 (1.7)	1.6 (1.2)	2.5 (1.5)	3.8 (1.8)
Fruits (serving/day)	1.1 (0.7)	2.2 (1.2)	3.0 (1.2)	1.4 (1.0)	2.0 (1.2)	2.6 (1.3)
Legumes (servings/day)	0.7 (0.4)	1.1 (0.7)	1.7 (0.8)	0.8 (0.7)	1.1 (0.8)	1.4 (0.8)
Nuts and seeds (serving/day)	0.1(0.2)	0.2 (0.3)	0.3 (0.4)	0.1 (0.2)	0.2 (0.3)	0.3 (0.4)
Eggs (servings/day)	0.1 (0.2)	0.2 (0.2)	0.4 (0.3)	0.2 (0.2)	0.2 (0.2)	0.3 (0.3)
Dairy products (serving/day)	0.1 (0.2)	0.3 (0.3)	0.4 (0.4)	0.2 (0.3)	0.2 (0.3)	0.3(0.3)
Animal meat (serving/day)	0.9 (0.5)	1.4 (0.6)	1.9 (0.7)	1.0 (0.6)	1.4 (0.7)	1.6 (0.7)
Sugar sweetened beverages (serving/day)	0.5 (0.4)	0.7 (0.5)	0.8 (0.6)	0.6 (0.5)	0.6 (0.5)	0.6 (0.5)
Sweets and deserts (serving/day)	0.3 (0.3)	0.5 (0.4)	0.6 (0.5)	0.4 (0.4)	0.5 (0.4)	0.5 (0.5)

Abbreviations: BMI, body mass index; MDD‐W, minimum dietary diversity for women; PDQS, Prime Diet Quality Score.

^a^ MDD‐W has a possible range of 0–10.

^b^ PDQS has a possible range of 0–42.

^c^ History of prior complications was defined as reporting any of the following: cardiovascular disease, high blood pressure, diabetes, weight loss in previous year, and ever having a low‐birth‐weight baby or nonlive birth if nonprimigravida.

^d^ Major nutrient intakes were presented as % of total energy intake.

**Table 2:** Summary on MDD‐W and intakes from individual MDD‐W food groups (*n *= 1190)

Food groups	≥1 time per day, *n* (%)
Starchy staples	1181 (99.2)
Pulses, beans, peas, lentils	553 (46.5)
Nuts, seeds	43 (3.6)
Dairy	120 (10.1)
Meat, poultry, fish	797 (67.0)
Eggs	67 (5.6)
Dark green‐leaf vegetables	320 (26.9)
Other vitamin A rich fruits and vegetables	482 (40.5)
Other vegetables	717 (60.3)
Other fruits	732 (61.5)
Meeting diversity (MDD‐W ≥5), *n* (%)	550 (46.2)
Overall mean (SD)	4.2 (1.9)
Correlation with energy intake^a^	0.72
Correlation with PDQS^a^	0.52

Abbreviations: MDD‐W, minimum dietary diversity for women; PDQS, Prime Diet Quality Score.

^a^ Spearman correlation coefficient was presented.

With respect to baseline population characteristics, women with higher MDD‐W were more likely to have longer education, skilled occupation, and a history of prior complications (Table 1). Women with higher MDD‐W were more likely to have higher energy intake, higher percentages of energy from protein and fat, and lower percentage of energy from carbohydrate. Higher MDD‐W was correlated with higher intakes of major food groups, including both healthy and unhealthy ones (Table 1).

In the main analyses on MDD‐W over the entire sample, overall, we did not observe evidence of association with any of the GWG outcomes that we examined, including inadequate GWG, excessive GWG, or inappropriate GWG. Similarly, no association was observed for any of the birth outcomes that we examined, including LBW, SGA, LGA, or preterm birth (Table 3). Similar results were observed after excluding women with a history of prior complications (Table 3). Alternatively modeling MDD‐W with binary levels provided consistent findings (Table S2).

**Table 3:** Associations between MDD‐W and GWG and adverse birth outcomes, overall (*n *= 1190) and excluding women with prior complications (*n *= 1004)

	MDD‐W			
	Continuous per SD	Tertile 1 (1, 3)	Tertile 2 (4, 5)	Tertile 3 (6, 9)
*GWG‐related outcomes*	RR, 95% CI^a^			
Inadequate GWG				
Overall (*n *= 502, 42.2%)^b^	1.02 (0.95–1.09)	Ref (RR = 1.00)	1.08 (0.92–1.27)	1.12 (0.95–1.33)
Excluding prior complications (*n* = 425, 42.3%)	1.01 (0.94–1.09)	Ref (RR = 1.00)	1.06 (0.89–1.27)	1.11 (0.92–1.33)
Excessive GWG				
Overall (*n *= 426, 35.8%)	0.96 (0.89–1.04)	Ref (RR = 1.00)	0.88 (0.73–1.05)	0.90 (0.74–1.09)
Excluding prior complications (*n* = 365, 36.4%)	0.96 (0.88–1.04)	Ref (RR = 1.00)	0.90 (0.74–1.10)	0.91 (0.74–1.13)
Inappropriate GWG^c^				
Overall (*n *= 928, 78.0%)	0.99 (0.96–1.02)	Ref (RR = 1.00)	0.98 (0.91–1.06)	1.01 (0.94–1.09)
Excluding prior complications (*n* = 790, 78.7%)	0.99 (0.96–1.02)	Ref (RR = 1.00)	0.99 (0.91–1.07)	1.01 (0.93–1.10)
*Birth outcomes*				
LBW^d^				
Overall (*n *= 92, 7.7%)	0.92 (0.74–1.15)	Ref (OR = 1.00)	1.13 (0.68–1.88)	0.75 (0.68–1.88)
Excluding prior complications (*n* = 73, 7.3%)	0.91 (0.71–1.17)	Ref (OR = 1.00)	1.07 (0.61–1.88)	0.69 (0.36–1.35)
SGA				
Overall (*n *= 198, 16.6%)	0.94 (0.84–1.06)	Ref (RR = 1.00)	0.89 (0.66–1.20)	0.86 (0.63–1.18)
Excluding prior complications (*n* = 159, 15.8%)	0.91 (0.79–1.05)	Ref (RR = 1.00)	0.80 (0.57–1.12)	0.81 (0.58–1.15)
LGA				
Overall (*n *= 125, 10.5%)	1.02 (0.86–1.21)	Ref (RR = 1.00)	1.02 (0.69–1.49)	0.93 (0.60–1.46)
Excluding prior complications (*n* = 107, 10.7%)	1.01 (0.84–1.22)	Ref (RR = 1.00)	0.94 (0.62–1.43)	0.95 (0.59–1.53)
Preterm birth^e^				
Overall (*n *= 183, 15.4%)	1.08 (0.94–1.24)	Ref (RR = 1.00)	1.10 (0.81–1.50)	1.09 (0.77–1.54)
Excluding prior complications (*n* = 151, 15.0%)	1.07 (0.92–1.24)	Ref (RR = 1.00)	1.03 (0.73–1.44)	1.08 (0.73–1.58)

Abbreviations: BMI, body mass index; CI, confidence interval; GWG, gestational weight gain; LBW, low birth weight; LGA, large for gestational age; MDD‐W, Minimum Dietary Diversity for Women; OR, odds ratio; RR, risk ratio; SGA, small for gestational age.

^a^ Multivariate model was adjusted for age (years), baseline BMI (kg/m^2^), gestational age at baseline (weeks), season (dry [December–March], long rains [April–May], harvest [June–September], short rains [October–November]), primigravida status (yes, no), marital status (married or cohabitating, other), treatment status (yes, no), education (0–4, 5–7, 8–11, >11 years), occupation (unemployed, unskilled/informal, skilled, other), and history of prior complications (any past complication in cardiovascular disease, high blood pressure, diabetes, weight loss in previous year, or ever having a low birth weight baby or nonlive birth among nonprimigravida).

^b^ Number of events (%) was presented.

^c^ Inappropriate GWG was defined as either inadequate or excessive GWG according to the Institute of Medicine guidelines.

^d^ Model for RR failed to converge. Adjusted OR and 95% CI from multivariable logistic regression model were presented.

^e^ There were four medically induced and 179 spontaneous preterm births.

### 3.2 PDQS, GWG, and birth outcomes

The mean score of PDQS was 23.3 (SD = 3.2) (Table 4). For the healthy food groups of PDQS, consumptions of vegetables (except cruciferous vegetables), fruits, legumes, fish, and vegetable oil were high, whereas consumptions of cruciferous vegetables, nuts, poultry, and eggs were low. For the unhealthy food groups, high consumptions of refined grains/baked foods and sugar‐sweetened beverages were observed. PDQS was correlated with energy intake (Spearman *r* = 0.39) but to a less extent compared to the correlation between MDD‐W and energy intake (Tables 2 and 4).

**Table 4:** Summary on PDQS and intakes from PDQS individual food groups (*n *= 1190)

	0–1 serving per week, *n* (%)	2–3 servings per week, *n* (%)	≥4 serving per week, *n* (%)
Healthy food groups			
Dark green vegetables	244 (20.5%)	329 (27.7%)	617 (51.9%)
Cruciferous vegetables	828 (69.6%)	252 (21.2%)	110 (9.2%)
Carrots and other vitamin‐A rich vegetables	476 (40.0%)	354 (29.7%)	360 (30.3%)
Other vegetables	107 (9.0%)	105 (8.8%)	978 (82.2%)
Whole citrus fruits	486 (40.8%)	295 (24.8%)	409 (34.4%)
Other fruits	40 (3.4%)	113 (9.5%)	1037 (87.1%)
Legumes	76 (6.4%)	189 (15.9%)	925 (77.7%)
Nuts	782 (65.7%)	303 (25.5%)	105 (8.8%)
Poultry	854 (71.2%)	315 (26.5%)	21 (1.8%)
Fish	151 (12.7%)	290 (24.4%)	749 (62.9%)
Eggs	722 (60.7%)	392 (32.9%)	76 (6.4%)
Whole grains	426 (35.8%)	412 (34.6%)	352 (29.6%)
Vegetable oil	95 (8.0%)	184 (15.5%)	911 (76.6%)
Low‐fat dairy	1190 (100%)	0	0
Unhealthy food groups			
Potatoes	580 (48.7%)	520 (43.7%)	90 (7.6%)
Red meat	281 (23.6%)	623 (52.4%)	286 (24.0%)
Processed meat	1190 (100%)	0	0
Refined grains and baked goods	11 (0.9%)	5 (0.4%)	1174 (98.7%)
Sugar‐sweetened beverages	281 (23.6%)	313 (26.3%)	596 (50.1%)
Fried food not from home	457 (38.4%)	333 (28.0%)	400 (33.6%)
Deserts and ice cream	397 (33.4%)	357 (30.0%)	436 (36.4%)
Overall mean (SD)		23.3 (3.2)	
Correlation with energy intake^a^		0.39	
Correlation with MDD‐W^a^		0.52	

Abbreviations: MDD‐W, minimum dietary diversity for women; PDQS, Prime Diet Quality Score.

^a^ Spearman correlation coefficient was presented.

With respect to the baseline population characteristics, women with higher PDQS were more likely to have a history of prior complications (Table 1). Women with higher PDQS were more likely to have higher total energy intake and slightly higher intake of protein. While higher intakes of major food groups, regardless of the food quality, were observed in women with higher MDD‐W, only higher intakes of healthy foods were observed for women with higher PDQS (Table 1).

In the analyses examining associations between PDQS and GWG in the entire sample, compared to the lowest tertile, women in the highest tertile group had borderline lower risk of inappropriate GWG (i.e., either below or above the recommended range) (RR = 0.93, 95% CI: 0.87–1.00) (Table 5). Risks of inadequate or excessive GWG did not significantly differ across the three tertile groups, respectively. Results excluding women with a history of complications showed similar findings.

**Table 5:** Associations between PDQS and GWG and adverse birth outcomes, overall (*n *= 1190) and excluding women with prior complications (*n *= 1004)

	PDQS			
	Continuous per SD			
	RR (95% CI)^a^	Tertile 1 (10, 21)	Tertile 2 (22, 24)	Tertile 3 (25, 31)
*GWG‐related outcomes*				
Inadequate GWG				
Overall (*n *= 502, 42.2%)^b^	0.97 (0.90–1.03)	Ref (RR = 1.00)	1.01 (0.85–1.18)	0.93 (0.79–1.10)
Excluding prior complications (*n *= 425, 42.3%)	0.98 (0.91–1.05)	Ref (RR = 1.00)	1.02 (0.86–1.22)	0.93 (0.77–1.12)
Excessive GWG				
Overall (*n *= 426, 35.8%)	1.00 90.92–1.07)	Ref (RR = 1.00)	0.91 (0.75–1.10)	0.95 (0.78–1.14)
Excluding prior complications (*n *= 365, 36.4%)	0.99 (0.91–1.07)	Ref (RR = 1.00)	0.92 (0.75–1.12)	0.94 (0.77–1.16)
Inappropriate GWG^c^				
Overall (*n *= 928, 78.0%)	0.98 (0.95–1.01)	Ref (RR = 1.00)	0.96 (0.89–1.03)	0.93 (0.87–1.00)
Excluding prior complications (*n *= 790, 78.7%)	0.98 (0.95–1.01)	Ref (RR = 1.00)	0.97 (0.90–1.05)	0.93 (0.86–1.01)
*Birth outcomes*				
LBW^d^				
Overall (*n *= 92, 7.7%)	0.84 (0.68–1.05)	Ref (OR = 1.00)	0.67 (0.40–1.13)	0.62 (0.36–1.05)
Excluding prior complications (*n *= 73, 7.3%)	0.77 (0.60–0.99)	Ref (OR = 1.00)	0.55 (0.31–0.99)	0.52 (0.29–0.94)
SGA				
Overall (*n *= 198, 16.6%)	0.95 (0.84–1.07)	Ref (RR = 1.00)	0.93 (0.69–1.26)	0.83 (0.61–1.13)
Excluding prior complications (*n *= 159, 15.8%)	0.97 (0.85–1.11)	Ref (RR = 1.00)	1.00 (0.71–1.40)	0.87 (0.62–1.24)
LGA				
Overall (*n *= 125, 10.5%)	0.99 (0.84–1.16)	Ref (RR = 1.00)	0.95 (0.63–1.44)	1.02 (0.68–1.53)
Excluding prior complications (*n *= 107, 10.7%)	0.96 (0.80–1.14)	Ref (RR = 1.00)	0.91 (0.59–1.40)	0.96 (0.63–1.48)
Preterm birth^e^				
Overall (*n *= 183, 15.4%)	0.97 (0.85–1.11)	Ref (RR = 1.00)	0.72 (0.51–1.00)	0.90 (0.66–1.23)
Excluding prior complications (*n *= 151, 15.0%)	0.96 (0.83–1.10)	Ref (RR = 1.00)	0.69 (0.48–0.99)	0.87 (0.62–1.22)

Abbreviations: BMI, body mass index; CI, confidence interval; GWG, gestational weight gain; LBW, low birth weight; LGA, large for gestational age; OR, odds ratio; PDQS, Prime Diet Quality Score; RR, risk ratio; SGA, small for gestational age.

^a^ Multivariate model adjusted for age (years), baseline BMI (kg/m^2^), gestational age at baseline (weeks), season (dry [December–March], long rains [April–May], harvest [June–September], short rains [October–November), primigravida status (yes, no), marital status (married or cohabitating, other), treatment status (yes, no), education (0–4, 5–7, 8–11, >11 years), occupation (unemployed, unskilled/informal, skilled, other), and history of prior complications (any past complication in cardiovascular disease, high blood pressure, diabetes, weight loss in previous year, or ever having a low birth weight baby or nonlive birth among nonprimigravida).

^b^ Number of events (%) was presented.

^c^ Inappropriate GWG was defined as either inadequate or excessive GWG according to the Institute of Medicine guidelines.

^d^ Model for RR failed to converge. Adjusted OR and 95% CI from multivariable logistic regression model were presented.

^e^ There were 4 medically induced and 179 spontaneous preterm births.

In the analyses examining PDQS and adverse birth outcomes, in the entire sample, women in the middle tertile of PDQS had borderline lower risk of preterm birth compared to women in the lowest tertile group (RR = 0.72, 95% CI: 0.51–1.00), while those in the highest tertile had reduced risk, although the result was not statistically significant (RR = 0.90, 95% CI: 0.60–1.23). After excluding women with prior complications, compared to the lowest tertile group, lower risk of LBW was observed in groups with higher PDQS (middle tertile: RR = 0.55, 95% CI: 0.31–0.99, highest tertile: RR = 0.52, 95% CI: 0.29–0.94; continuous per SD: RR = 0.77, 95% CI: 0.60–0.99). Compared to the lowest tertile group, lower risks of preterm birth were observed in groups with higher PDQS (middle tertile: RR = 0.69, 95% CI: 0.48–0.99; highest tertile: RR = 0.87, 95% CI: 0.62–1.22) (Table 5).

## 4 DISCUSSION

This study prospectively examined maternal dietary diversity and diet quality using MDD‐W and PDQS, respectively, and their associations with inappropriate GWG and adverse birth outcomes in a healthy pregnancy cohort in urban Tanzania. MDD‐W was generally not associated with risk of GWG or adverse birth outcomes, whereas higher PDQS was associated with lower risk of inappropriate GWG and lower risks of LBW and preterm birth, highlighting the importance of maternal diet quality as a potential modifiable factor for preventing inappropriate GWG and adverse infant outcomes among Tanzanian women.

MDD‐W was developed as a measure to assess overall dietary diversity in LMIC settings, and it has been previously validated to be correlated with nutrient adequacy of 11 micronutrients, including folate, key vitamins, calcium, iron, and zinc (Arimond et al., 2010; FAO, 2021). PDQS was developed to assess overall diet quality by taking into account of intakes from both healthy and unhealthy foods, and it has been widely applied in studies conducted in developed settings (Fung et al., 2018; Gicevic et al., 2018). Summary characteristics of MDD‐W and PDQS in this study were consistent with those reported from earlier SSA pregnancy studies (mean MDD‐W ranging between 4.0 and 6.0, % meeting diversity ranging between 40% and 60%; median PQDS = 19) (Huang et al., 2018; Lauer et al., 2020; Madzorera et al., 2020; Nsereko et al., 2020), except the earlier study by Madzorera et al. in Tanzania where a lower percentage of MDD‐W ≥ 5 was reported (2.8%) (Madzorera et al., 2020), supporting the overall validity of our findings.

In this study, we did not observe any association between MDD‐W and GWG, but higher PDQS was associated with lower risk of inappropriate GWG. While no studies in SSA have examined MDD‐W or PDQS with GWG, a longitudinal study in urban South Africa (*n *= 538) examined western, traditional, and mixed maternal dietary patterns; the authors reported that increased intakes of a traditional diet pattern with high in whole grains, legumes, vegetables, and traditional meats and low intakes of refined grains, sugar, and fats, were associated with lower risk of excessive GWG (odds ratio [OR] = 0.81, *p* = 0.006) (Wrottesley et al., 2017), supporting the role of high‐quality maternal diets in supporting optimal GWG in African population. Studies conducted in developed settings also reported similar conclusions (Guilloty et al., 2015; Itani et al., 2020; Stuebe et al., 2009; Tielemans et al., 2015; Uusitalo et al., 2009). For this present study, given its enrollment criteria that entailed excluding women with anemia at baseline and the study setting in urban Eastern SSA, women in this study were in general well‐nourished with secure food access and less concern of under‐nutrition or suboptimal dietary diversity. Since PDQS considered both quantity and quality of the diet, it might be more useful in characterizing maternal dietary patterns in this well‐nourished Tanzanian population.

We did not observe any association between MDD‐W and the birth outcomes that we examined, including LBW, SGA, LGA and preterm birth. There were a few African studies that examined MDD‐W or other diversity metrics with birth outcomes. Madzorera et al. prospectively examined MDD‐W in a HIV‐negative pregnancy cohort in Tanzania (*n* = 7553), using repeated 24‐h recalls; with a low percentage of MDD‐W ≥ 5 (2.8%), they found that higher MDD‐W was associated with lower risk of SGA (highest quintile vs. lowest quintile: OR = 0.74, 95% CI: 0.62–0.89) (Madzorera et al., 2020). Another prospective study in Rwanda (*n *= 367; percentage of MDD‐W ≥ 5: 50%) reported that lower MDD‐W was associated with higher risk of preterm birth (MDD‐W < 5 vs. MDD‐W ≥ 5: OR = 3.94, 95% CI: 1.57–9.91) (Nsereko et al., 2020). Other African studies using different metrics assessing dietary diversity also reported associations between higher dietary diversity and lower risks of LBW and preterm birth (Saaka, 2013; Zerfu et al., 2016). Compared to these earlier studies, the null associations observed in our study could be due to differences in population characteristics, timing of maternal diet assessment, and different dietary assessment method.

We observed that higher PDQS was associated with lower risks of preterm birth (middle tertile group only) and LBW. The earlier Tanzania study by Madzorera et al. also examined PDQS; they observed protective associations between higher PDQS and lower risks of preterm birth, LBW, and fetal loss (highest quintile vs. lowest quintile: RR = 0.55, 0.53, and 0.53, respectively; *p*‐trend < 0.001) (Madzorera et al., 2020).

The observed patterns of associations differed between MDD‐W and PDQS for GWG and birth outcomes, which might be explained by the fact that these two scores measure different aspects of diet. MDD‐W was developed to mainly assess micronutrient adequacy (FAO, 2021); a diet with a greater variety of foods would likely result in a higher MDD‐W, irrespective of food quality. PDQS, on the other hand, can differentiate healthy and unhealthy foods by negatively scoring consumption of foods that are associated with obesity, inflammation, and insulin resistance (Fung et al., 2018; Madzorera et al., 2020). The overall null associations observed for MDD‐W could be due to relatively good access to foods among this urban pregnancy cohort, thus limiting its utility in the present study setting. Overall, these findings support the utilization of PDQS assessing maternal diet in urban SSA settings, when used in conjunction with either 24‐h recalls or FFQs, and they support the importance of high‐quality and balanced maternal diets on preventing adverse birth outcomes among Tanzanian women.

Dietary diversity is a key component for a healthy maternal diet. There are several key micronutrients involved in immune system functioning and tissue growth, including folate, zinc, iron, and key vitamins (Gernand et al., 2016; Mousa et al., 2019). Malnutrition due to micronutrient deficiency negatively influences immune system, thus increasing risks of maternal, placental, and fetal inflammation from infection (Fawzi et al., 2007; Goldenberg, 2003); it also influences oxidative metabolism that leads to pathological stress and hormonal imbalance, affecting maternal‐placental functioning and epigenetic programming of the fetus (Gernand et al., 2016). Maternal diets of high quality provide adequate high‐quality macronutrients, such as protein (Kramer & Kakuma, 2003) and healthy fatty acids (Abu‐Saad & Fraser, 2010; Larqué et al., 2012), which are important for immune functioning, optimal GWG, and fetal growth (Mennitti et al., 2015; Mousa et al., 2019). A high‐quality maternal diet also implies limited consumptions of high‐energy foods with low nutrient density, thus lowering risks of excessive GWG and obesity‐related pregnancy events (Guelinckx et al., 2008; Zhang et al., 2006). A poor maternal diet with suboptimal diversity and quality would fail to meet the nutrition required for both mother and the fetus, thus leading to higher risks of in‐pregnancy complications and adverse birth outcomes.

In this overall healthy and well‐nourished pregnancy cohort in urban SSA, sufficient intakes of energy and key macronutrients were observed. However, we also observed low consumption of proteins and healthy fats that were important for maternal health and fetal development. In addition, intakes of refined grains and sugar‐contained foods were high in this urban African cohort, supporting the recent nutrition transition to a more Westernized diet high in unhealthy fats, sugar, and processed foods observed in some SSA countries (Lindsay et al., 2012; Wrottesley et al., 2017), concerning increasing trends in obesity and possibly obesity‐related pregnancy complications, such as LGA and gestational diabetes with long‐term health consequences (Muche et al., 2019; Popkin et al., 2012). In addition to meeting dietary diversity, choice of high‐quality foods, moderation, and energy balance should also be advised during pregnancy for optimal pregnancy outcomes for women in urban SSA. Overall, compared to the earlier SSA studies, our findings provide additional insights on the current nutritional gaps on maternal diet among Tanzanian women, and they contribute to knowledge of the significance of healthy diets emphasized in the UNICEF global strategies for nutrition in the 2020–2030 decade (UNICEF, 2020). This study calls for continued advocation of right messaging on high‐quality maternal diets with well‐balanced food choices, through prenatal counseling, for pregnant women in SSA.

Strengths of this study include the prospective study design examining maternal diet and pregnancy outcomes among a well‐nourished African population, detailed dietary information collected by FFQs, well‐characterized GWG with repeated weight measures, and sufficient covariate adjustment. However, this study has several limitations. First, diet was assessed by the FFQ only once at the study baseline. Thus, it may only represent early‐pregnancy dietary habits. However, since FFQ aimed to assess long‐term dietary pattern compared to other dietary assessments, and maternal diet was more sensitive to external factors (e.g., SES, food availability due to seasonal change) rather than timing of the pregnancy (Fowles & Fowles, 2008), our results may be generalized to the overall dietary diversity and quality over the course of pregnancy. Second, diet was likely to be reported in FFQ with errors. Additionally, FFQ may have over‐estimated dietary diversity compared to 24‐h recall employed in the previous SSA studies. Nevertheless, since diet was assessed prospectively before the outcomes, any misclassification on the exposure would be non‐differential with respect to the outcomes, thus attenuating the associations towards the null. Thirdly, similar to other studies conducted in LMICs, gestational age was estimated based on the last menstrual period (LMP). Thus, errors on LMP reporting would lead to nondifferential misclassification of outcomes related to gestational age. In addition, similar to other observational studies, we could not rule out the possibility of residential confounding. Finally, our results can only be generalized to SSA populations with similar population characteristics.

In conclusion, this study provides an updated profile of maternal diet in urban SSA and highlight the importance of maternal diet quality on optimal GWG and birth outcomes. Local operational research is needed to develop effective strategies to assess and implement healthy maternal diets for pregnant women in real practice.

## CONFLICT OF INTERESTS

The authors declare that there are no conflict of interests.

## ETHICS STATEMENT

The original trial was ethically approved by the Harvard School of Public Health Human Subjects Committee, the Muhimbili University of Health and Allied Sciences Research and Publications Committee, and Tanzania's National Institute for Medical Research. Written informed consent was obtained from all women for their participation in the study.

## AUTHOR CONTRIBUTIONS

Jiaxi Yang, Wafaie W. Fawzi, Deirdre K. Tobias, Janet W. Rich‐Edwards, and Molin Wang designed analysis plan. Jiaxi Yang conducted statistical analysis and drafted the manuscript. Molin Wang supervised statistical analysis. Isabel Madzorera and Ajibola I. Abioye provided programming support. Wafaie W. Fawzi was the principal investigator of the original trial study. All authors read and approved the final manuscript.

## Supporting information

Supporting information.

## Data Availability

The data set analyzed during the current study is not publicly available due to regulatory obligations of the collaborating institutions but may be available from the corresponding author on reasonable request.
