# Supplementary material for: Dietary diversity and diet quality with gestational weight gain and adverse birth outcomes, results from a prospective pregnancy cohort study in urban Tanzania
Source: Matern Child Nutr. 2021 Dec 14;18(2):e13300. doi: 10.1111/mcn.13300 (PMC8932689; doi:10.1111/mcn.13300)

**Supporting Information**

Supplement Table 1. Details on derivations of serving/day from food frequency questionnaire administered at study baseline

Supplement Table 2. Associations between meeting MDD-W diversity (MDD-W≥5) and GWG and adverse birth outcomes (n=1,190)

Supplement Figure 1. Directed acyclic graph for maternal diet, GWG, and birth outcomes

Footnote: Maternal diet influences birth outcomes with GWG as a potential mediator. GWG may influence birth outcomes through other upstream mechanisms, where maternal diet may act as an effect modifier.

Supplementary Figure 2. Underlying mechanisms between maternal diet quality, GWG, and birth outcomes

**Supplement Table 1.** Details on derivations of serving/day from food frequency questionnaire administered at study baseline

| Frequency reported on FFQ | Corresponding servings/day |
| --- | --- |
| Never | 0 serving/day |
| 1-3 times per month | 2 servings/30 days= 0.07 serving /day |
| 1 time per week | 1 serving/7 days=0.14 serving/day |
| 2-4 times per week | 3 servings/7 days=0.43 serving/day |
| 5-6 times per week | 5.5 servings/7 days=0.79 serving/day |
| 1 time per day | 1 servings/day |
| 2-3 times per day | 2.5 servings/day |
| 4-5 times per day | 4.5 servings/day |
| 6 time per day | 6 servings/day |

**Supplement Table 2:** Associations between meeting MDD-W diversity (MDD-W≥5) and GWG and adverse birth outcomes (n=1,190)

|  | **Status of meeting MDD-W criteria** | |
| --- | --- | --- |
|  | Not meeting MDD-W (n=640, 53.8%) | Meeting MDD-W  (n=550, 46.2%) |
|  | RR, 95% CI† | |
| **GWG-related outcomes** |  |  |
| Inadequate GWG (n=502, 42.2%)‡ | Ref (RR=1.00) | 0.94 (0.82-1.08) |
| Excessive GWG (n=426, 35.8%) | Ref (RR=1.00) | 0.99 (0.85-1.15) |
| Inappropriate GWG (n=928, 78.0%)§ | Ref (RR=1.00) | 0.96 (0.91-1.03) |
| **Birth outcomes** |  |  |
| LBW (n=92, 7.7%)¶ | Ref (OR=1.00) | 0.79 (0.50-1.23) |
| SGA (n=198, 16.6%) | Ref (RR=1.00) | 0.87 (0.67-1.11) |
| LGA (n=125, 10.5%) | Ref (RR=1.00) | 0.91 (0.64-1.29) |
| Preterm birth (n=183, 15.4%) | Ref (RR=1.00) | 1.11 (0.86-1.45) |

Abbreviations: BMI, body mass index. CI, confidence interval. GWG, gestational weight gain. LBW, low birth weight. LGA, large for gestational age. MDD-W, Minimum Dietary Diversity for Women. OR, odds ratio. RR, risk ratio. SD, standard deviation. SGA, small for gestational age.

† Multivariate model adjusted for age (years), baseline BMI (kg/m^2^), gestational age at baseline (weeks), season (dry [December-March], long rains [April-May], harvest [June-September], short rains [October-November]), primigravida status (yes, no), marital status (married or cohabitating, other), treatment status (yes, no), education (0-4 years, 5-7 years, 8-11 years, >11 years), occupation (unemployed, unskilled/informal, skilled, other), and history of prior complications (any past complication in cardiovascular disease, high blood pressure, diabetes, weight loss in previous year, or ever having a low birth weight baby or non-live birth among non-primigravida).

‡ Number of events (%) was presented.

§ Inappropriate GWG was defined as either inadequate or excessive GWG according to the Institute of Medicine guidelines.

¶ Model for RR failed to converge. Adjusted OR and 95% CI from multivariable logistic regression were presented.

**Supplement Figure 1.** Directed acyclic graph for maternal diet, GWG, and birth outcomes


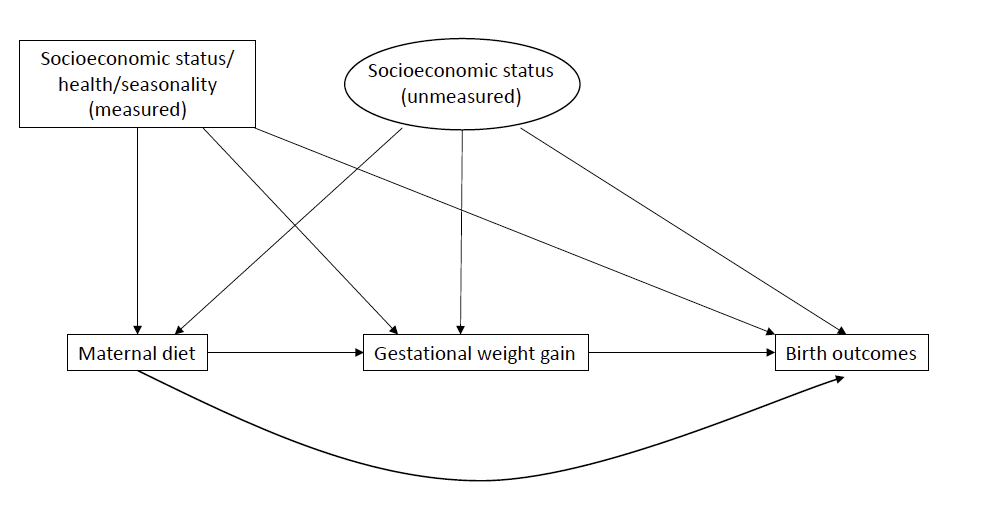


**Supplementary Figure 2.** Underlying mechanisms between maternal diet quality, GWG, and birth outcomes


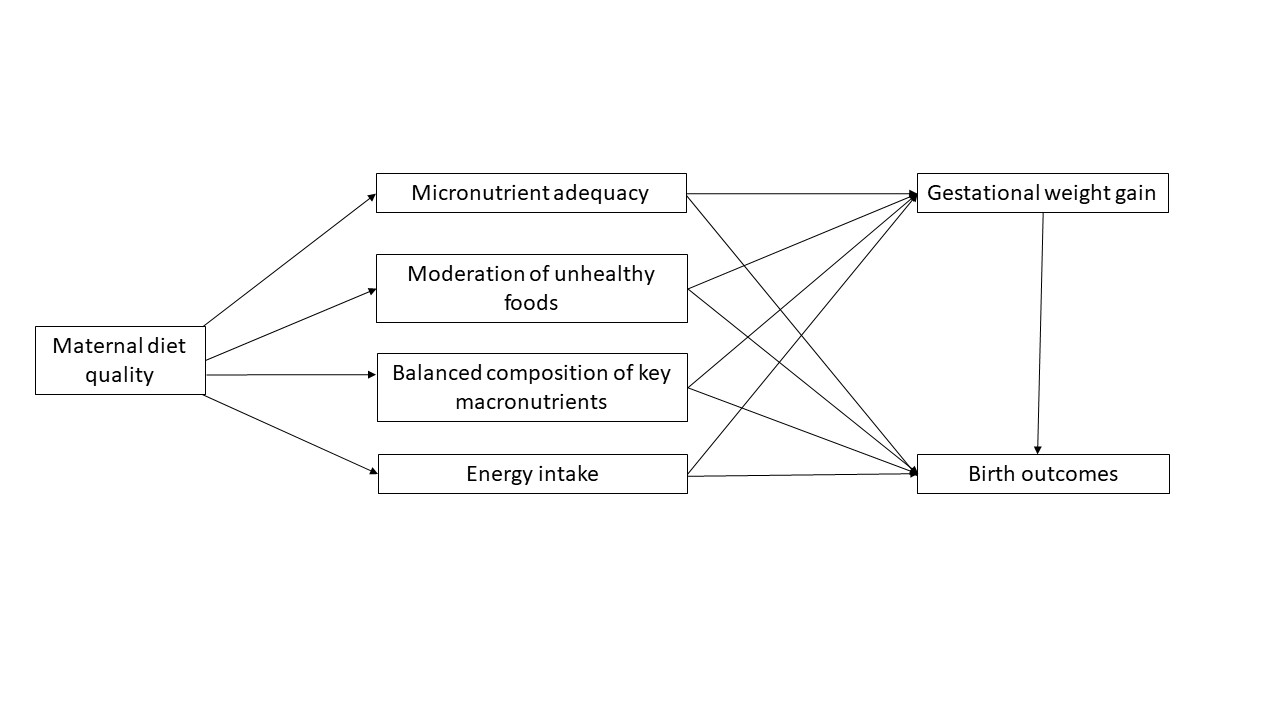

Supplement: Supplementary file 1 — Supporting information. [file MCN-18-e13300-s001.docx]
